# A Method of Well-Spread Pachytene Chromosome Preparations for Plant Species with Large Genomes Suitable for the Immunolocalization of Meiotic Proteins

**DOI:** 10.3390/mps8030054

**Published:** 2025-05-19

**Authors:** Natalya Kudryavtseva, Aleksey Ermolaev, Ludmila Khrustaleva

**Affiliations:** 1 All-Russian Research Institute of Agricultural Biotechnology, 42 Timiryazevskaya Str., 127550 Moscow, Russia; ermol-2012@yandex.ru (A.E.); khrustaleva@rgau-msha.ru (L.K.); 2 Center of Molecular Biotechnology, Russian State Agrarian University—Moscow Timiryazev Agricultural Academy, 49 Timiryazevskaya Str., 127550 Moscow, Russia

**Keywords:** pachytene, synaptonemal complex, *Allium fistulosum*, large genome, immunodetection, meiotic protein

## Abstract

Well-spread pachytene chromosomes are critical for studying the location of meiotic proteins along individual chromosomes. However, producing good spreads in species with large genomes is challenging due to the tangling of pachytene chromosomes. Existing protocols often fail to achieve proper separation of large chromosomes in spreads. Here, we describe in detail an improved protocol that ensures the effective separation of large pachytene chromosomes and demonstrates its suitability for protein immunodetection. To develop the protocol, pollen mother cells at the middle–late pachytene stage from *Allium fistulosum*, a species with a large genome and chromosomes, were used. The protocol involved three main steps: fixing anthers in Clark’s solution (ethanol–acetic acid, 3:1), digestion in an enzyme mixture, and gentle squashing in 45% acetic acid. A clear ZYP1 signal on all separated chromosomes was observed. The high quality of well-spread pachytene chromosomes obtained with the modified protocol allowed for the easy extraction of individual chromosomes for more precise detection and analysis of the proteins of interest.

## 1. Introduction

The preparation of well-spread chromosomes is a critical step for obtaining reliable results in molecular cytogenetic studies. Modern molecular cytogenetic methods, such as *in situ* hybridization (ISH) and protein immunodetection, require high-quality chromosome preparations. Plant pachytene chromosomes are 7–40 times longer than mitotic metaphase chromosomes, making individual chromosome pairing and synaptonemal complex (SC) formation challenging to analyze. Well-spread pachytene chromosomes for protein immunodetection have been reported for species with small genomes and chromosome sizes, such as *Arabidopsis arenosa* (2n = *2x* = 16, 150 Mb) [1,2], *Zea mays* (2n = *2x* = 20, 2500 Mb) [3], and *Solanum lycopersicum* (2n = *2x* = 24, 824 Mb) [4]. However, current methods do not yield well-spread pachytene chromosomes in species with large genomes. The tangled threads of pachytene chromosomes make it difficult to analyze the localization of meiotic proteins along individual chromosomes. This knowledge is critical for successful plant breeding, particularly in interspecific breeding programs analyzing synapsis and recombination at target site. Moreover, in some species, crossovers (COs) occur exclusively in specific chromosomal regions. For example, in barley, COs are localized in the distal regions [5], while in Welsh onion (*Allium fistulosum*), they are localized in the proximal region [6], limiting the genetic variability available to breeders. Studying these phenomena requires an analysis of meiotic protein distribution involved in recombination along individual SCs.

Various protocols for preparing plant pachytene chromosomes have been developed during studies of meiotic proteins using immunodetection techniques. One approach utilizes detergents to dissolve cell membranes and release chromatin onto slides [2,7,8,9,10,11,12,13,14,15,16,17,18]. Another method employs hypotonic solutions to induce cell swelling and burst [4,19,20]. A third technique involves squashing anthers in PBS, with or without detergents [21,22,23,24,25]. These approaches typically use formaldehyde as a fixative, which creates intermolecular cross-links between proteins and chromatin, preserving chromosome morphology under harsh conditions [26]. Alternatively, Clark’s fixative—an ethanol-based coagulation fixative—has been used to enhance antigen detection and improve meiotic chromosome separation applying spreading method for slide preparation [27]. In this protocol, the authors used a spread method for chromosome preparation that did not allow the distinction of individual pachytene chromosomes, even for species with small chromosomes, such as *Arabidopsis* and *Brassica*. Our previous work [6] introduced a dropping method for *Allium* meiotic chromosome preparation using Clark’s fixative, which proved effective for the immunodetection of certain meiotic proteins. However, this method did not produce well-spread pachytene chromosomes for analyzing protein location on individual chromosomes. Thus, currently, no established protocols allow for the preparation of well-spread large pachytene chromosomes suitable for immunudetection.

To make progress in this field, we describe a detailed, reliable protocol for preparing well-spread pachytene chromosomes from species with large genomes, optimized for meiotic protein immunodetection. The protocol was developed using pollen mother cells (PMCs) of *A. fistulosum* (2n = *2x* = 16, genome size 11,672 Mb), which possesses one of the largest plant chromosomes and serves as an excellent cytogenetic model. A single *A. fistulosum* chromosome contains approximately the same amount of DNA as the entire diploid genome of tomato (*S. lycopersicum*). At the pachytene stage, the average synaptonemal complex (SC) length per bivalent is 90 μm (total SC length 719 μm) in *A. fistulosum* [28], compared to 18 μm (total 230 μm) in tomato [29]. Our method employs Clark’s fixative with gentle squashing in 45% acetic acid to achieve sufficient chromosome spreading. We conducted a comparative analysis of 45% versus 60% acetic acid for squashing. The protocol includes troubleshooting guidance and identifies critical steps in meiotic chromosome preparation. Using this method, we successfully visualized the ZYP1 protein (a transverse filament of SC) on *A. fistulosum* pachytene chromosomes and achieved complete telomere-to-telomere tracing of individual chromosomes. We measured SC lengths for each bivalent and compared them with scanning electron microscopy (SEM) data from spread pachytene chromosomes [28]. The absence of chromosome stretching has been demonstrated in samples prepared using our protocol compared to spread SC.

## 2. Experimental Design

PMCs of *A. fistulosum* were used. Plants were cultivated in pots in the greenhouse under controlled conditions: a photoperiod of 16 hours of day/8 hours of night at a temperature of 22 °C and at 50% relative humidity.

To achieve well-spread preparations, the squashing of pachytene chromosomes in 45% acetic acid was applied instead of dropping cell suspensions on a slide.

Previous work [6] based on Clark’s ethanol fixation, demonstrates good results in experiments for the immunnodetection of some meiotic proteins. The meiotic protein immunodetection protocol was performed as described [6], with minor modifications. Briefly, slides were incubated with 1% blocking buffer for 30 min at room temperature, followed by incubation with rat anti-ZYP1 antibody (1:20 dilution in blocking buffer) for 18 h at 4 °C. After three washes in 1 × PBS containing 0.1% Tween-20 (10 min each at room temperature), slides were incubated with Alexa Fluor 488-conjugated goat anti-rat secondary antibody (Abcam, Cambridge, UK; 1:100 dilution in blocking buffer) for 3 h at 37 °C.

Individual pachytene chromosomes with a bright signal of continuous ZYP1 tracks were extracted using the Straighten plugin (https://imagej.net/ij/plugins/straighten.html, accessed on 1 March 2025) in ImageJ 1.54p software [30]. Image processing (cropping, scale bars and letters placement, and collage assembly) was performed using GIMP 2.10.38 (https://www.gimp.org/, accessed on 1 March 2025). All original uncropped microscopic images are available in the Supplementary Material.

To assess the effect of the squashing method on the length of chromosomes, a comparison of the lengths of squash and spread SCs was carried out. The SC length of individual chromosomes was measured using the DRAWID v0.26 software for karyotype analysis and ideogram construction [31] (detailed user manual could be found in GitHub repository https://github.com/Kirovez/DRAWID, accessed on 1 March 2025). DRAWID is a user-friendly, Java-based software program featuring an intuitive graphical user interface. The tool enables the measurement of centromere index, arm ratio, relative and absolute chromosome (or SC) length, signal and band positions, and size. The standard error (SE) for each chromosome was estimated using Microsoft Excel.

### 2.1. Materials and Equipment

#### 2.1.1. Consumables

- Petri dish;
- Pasteur pipettes;
- Dissecting needle straight;
- Dissecting needle lancet-shaped;
- Pipette tips 1000 μL;
- Superfrost microscope slides (without adhesion);
- 24 × 24 mm coverslips;
- Scalpel;
- Filter paper;
- Box filled with ice;
- Liquid nitrogen.

#### 2.1.2. Reagents

- Glacial acetic acid (CH_3_COOH);
- Sodium hydroxide (NaOH);
- Citric acid (Sigma-Aldrich Co., LLC, St. Louis, MO, USA, Cat. no.: C2404);
- Citric acid trisodium salt dihydrate (Panreac, Darmstadt, Germany, Cat. no.: 131655);
- Pectolyase from *Aspergillus japonicus* (Sigma-Aldrich Co., LLC, St. Louis, MO, USA, Cat. no.: P5936);
- Cellulase Onozuka R-10 (SERVA, Heidelberg, Germany, Cat. no.: 16419);
- Cytohelicase from *Helix pomatia* (Sigma-Aldrich Co., LLC, St. Louis, MO, USA, Cat. no.:C8274).

#### 2.1.3. Equipment

- Water bath (Miulab, Hangzhou, Zhejiang, China; Cat. no.: SWT-100);
- Light Microscope Axio Imager M2 (Carl Zeiss AB, Stockholm, Sweden; Cat. no.: 490020-0004-000).

## 3. Procedure of Well-Spread Pachytene Chromosome Preparation

### 3.1. Anther Selection at the Middle-Late Pachytene Stage and Fixation

1. Extract a single anther from the selected bud.
2. Place the extracted anther in a 60% acetic acid solution and gently squash it on a microscope slide.
3. Examine the squashed preparation under a light microscope to confirm the middle-late pachytene stage.
4. If the stage is confirmed, collect the remaining anthers from the same bud into a tube containing Clark’s fixative.
5. Leave the anthers in Clark’s fixative for 1 hour at room temperature.

PAUSE STEP:

1. Anthers can be used immediately for chromosome preparations or stored in Clark’s fixative at −20 °C before use (no more than 5 months).

### 3.2. Enzymatic Digestion: Duration: 2:30 h

1. Rinse the fixed anthers in water 3 times for 10 min each.
2. Rinse the anthers with citrate buffer for 5 min.
3. Transfer the anthers to the Petri dish with the enzyme mixture (50 μL of enzyme mixture per 10 anthers) for 120 min at 37 °C in a water bath.
4. Stop the enzyme reaction by transferring the Petri dish with anthers to ice.

CRITICAL STEP:

1. The anthers should be submerged at the bottom of the enzyme drop using a dissecting needle. Try not to damage the integrity of the anthers.
2. Conditions for proper enzyme treatment should be adapted for the species being analyzed (see our previous publication [32]).

### 3.3. Chromosome Squash Preparation—Duration: 10 min

**Figure 1:**
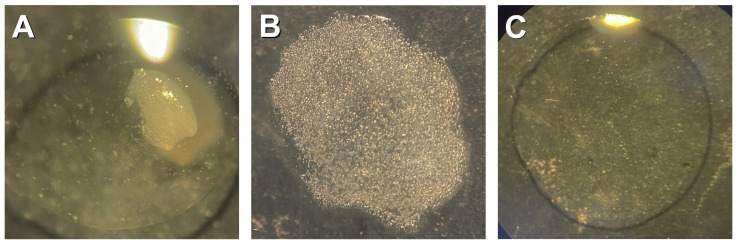
The preparation of PMC suspension: (**A**) an anther in a drop of enzyme mixture on a slide, (**B**) cell suspension after anther crushing, and (**C**) a transparent cell suspension after the addition of 45% acetic acid.

1. Carefully transfer one anther with a small drop of enzyme mixture to a slide using a lancet-shaped dissecting needle and crush the anther with a straight dissecting needle to obtain a fine cell suspension (Figure 1A,B).
2. Add a drop of 45% acetic acid to the slide and gently mix. The cell suspension should become transparent (Figure 1C).
3. Cover the cell suspension with a cover glass and gently tap on the entire area of the cover glass with a pipette tip.
4. Cover the preparation with filter paper and press lightly to remove excess liquid, avoiding any horizontal movement.
5. Freeze the slides in liquid nitrogen and remove the cover glass with a scalpel.
6. Dry the slides in the air.
7. The preparation can be used immediately for immunodetection.

CRITICAL STEP:

1. The anther disintegrates better in a small drop of liquid. Meanwhile, an insufficient amount of liquid can lead to rapid drying of the cell suspension on the slide. Be careful and attentive.
2. Excessive pressure on the coverslip results in strong and uneven stretching of the pachytene chromosomes.

## 4. Expected Results

**Figure 2:**
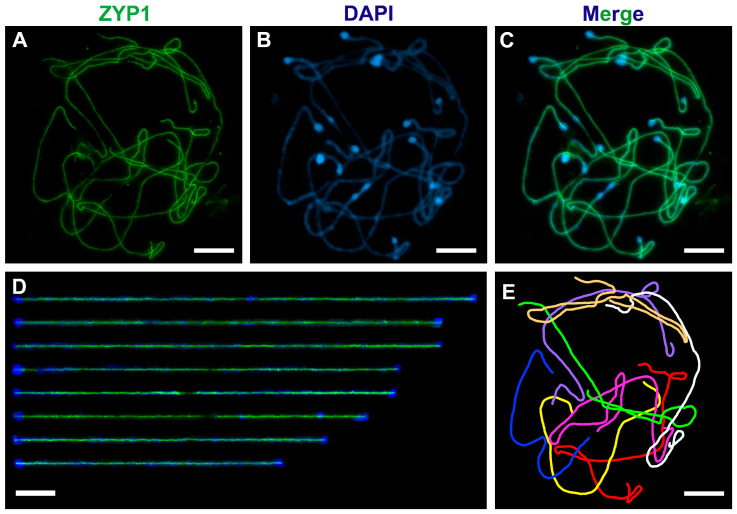
Immunodetection of the ZYP1 protein (green) in Clark’s fixed spreads (**A**–**C**). Pachytene chromosomes were extracted using the Straighten plugin (https://imagej.net/ij/plugins/straighten.html, accessed on 1 March 2025) in ImageJ 1.54p software [30] (**D**). A scheme in which each pachytene chromosome is labeled with a different color (**E**). Chromatin was stained with DAPI (blue). The scale bars represent 10 μm.

The application of Clark’s fixative combined with the sqashing technique yielded high-quality, well-spread pachytene chromosomes (Figure 2A–C). This ethanol-based fixative preserves protein antigenicity through precipitation [33]. Using our optimized protocol, we observed continuous ZYP1 signals along all chromosomes in over 90% of spreads (Figure 2A–C). Previously, successful the visualization of cohesin proteins (REC8, SCC3), synaptonemal complex proteins (ASY1, ZYP1), and recombination proteins (MLH1, MUS81) was achieved with Clark’s fixative [6,27]. However, RAD51 and DMC1 proteins involved in the repair of DNA double-strand breaks were not detected [27], emphasizing that this fixation method is not universally suitable for all types of protein detection.

We successfully extracted all pachytene chromosomes (Figure 2D,E) and measured the SC lengths for each chromosome across 28 cells (Table 1). The total mean SC length was 754.32 μm. Previously, Albini and Jones obtained well-spread pachytene chromosomes of *A. fistulosum* for SEM microscopy [28]. The overall mean SC length measured on our spreads was 5% greater than 719.98 µm obtained by Albini and Jones. Additionally, the relative lengths of SC for each chromosome ranked by their length were consistent with those reported by Albini and Jones (Table 1). Thus, proper treatment with the enzyme mixture and gentle squashing of the anthers in 45% acetic acid do not affect the morphology and stretching of the pachytene chromosomes.

### 4.1. Troubleshooting

#### 4.1.1. The Quality of the Chromosome Preparation Depends on the Cytoplasm Density

**Figure 3:**
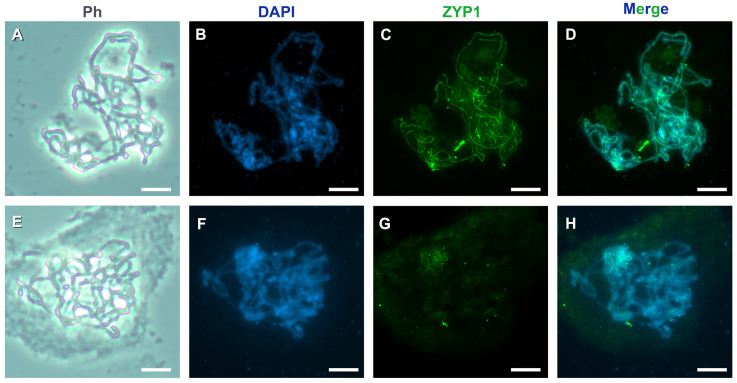
Visualization of the ZYP1 protein (green) on the *A. fistulosum* chromosomes at pachytene stage: cell with little cytoplasm (**A**–**D**) and cell with dense cytoplasm (**E**–**H**). Chromatin was stained with DAPI (blue). The scale bars represent 10 μm.

The cytoplasm affects the accessibility of antibodies to target proteins (Figure 3). The presence of a little cytoplasm on the chromosome preparations ensures the accessibility of antibodies to target proteins. Therefore, bright, continuous ZYP1 signals can be detected without high background levels (Figure 3A–D). In contrast, dense cytoplasm in pachytene chromosome preparations results in a high background levels and a decreased intensity of ZYP1 signals (Figure 3E–H). We recommend checking for cytoplasm on slides before performing immunodetection of proteins. If a dense cytoplasm is present on all chromosome preparations, the anther’s incubation time and the enzyme ratio in the mixture should be adjusted. The 60% acetic acid during cell squashing should not be employed to remove cytoplasm (see Section 4.1.2).

#### 4.1.2. The Concentration of Acetic Acid Affects the Degree of Chromosome Stretching and Spreading

**Figure 4:**
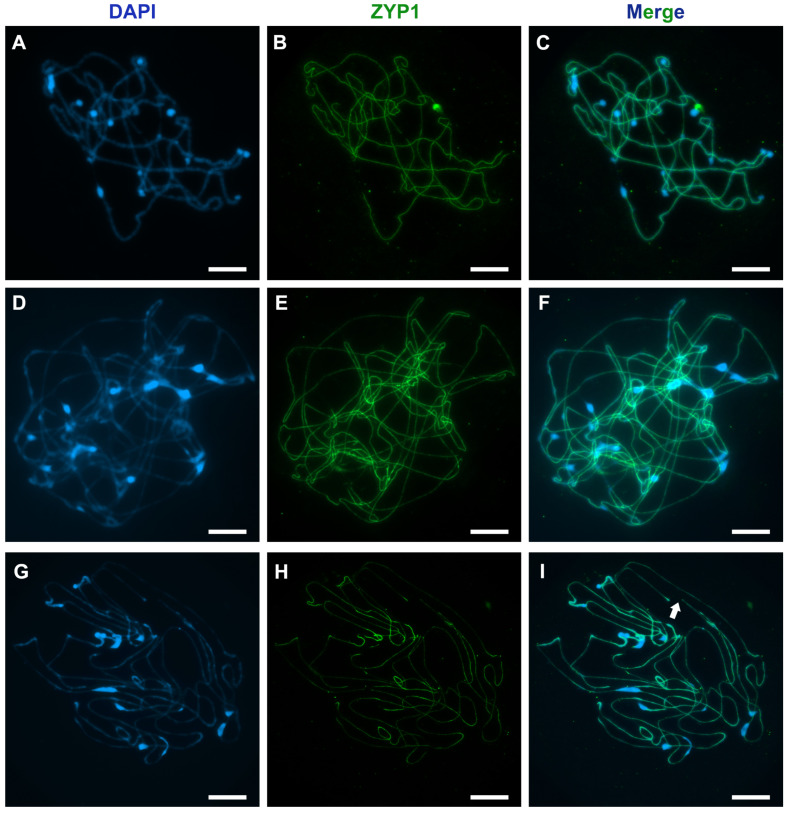
Immunodetection of ZYP1 protein (green) on *A. fistulosum* pachytene chromosomes after squashing of PMCs in 45% (**A**–**C**) and 60% (**D**–**I**) acetic acid. Arrows indicate areas where chromosomes broke during the squashing process. Chromatin was stained with DAPI. The scale bars represent 10 μm.

The concentration of acetic acid used during cell squashing significantly affects the stretching and spreading of pachytene chromosomes. A 45% acetic acid solution results in well-spread chromosomes, with little effect on the chromatin structure. (Figure 4A–C). In contrast, a 60% acetic acid solution causes excessive and uneven stretching (Figure 4D–F) and may lead to chromosome breakage (Figure 4G–I).

## 5. Reagent Setup

- Clark’s fixative: mix 3 volumes 96% ethanol and 1 volume glacial acetic acid. Use fresh solution.
- Citrate buffer: dissolve 0.558 g sodium citrate and 0.384 g citric acid in 100 mL distilled water. Adjust pH to 4.8 with NaOH (1 M). Sterilize and store at +4 °C.
- Enzyme mixture: dissolve 0.003 g pectolyase, 0.003 g cellulase and 0.003 g cytohelicase in 100 μL citrate buffer.
- 45% and 60% acetic acid solution in water.

## 6. Conclusions

In this study, we present the protocol for producing well-spread pachytene chromosomes for species with large genomes and chromosome sizes. The protocol comprises three key steps: fixation of anthers in Clark’s fixative, digestion with an enzyme mixture, and gentle squashing in 45% acetic acid. Immunodetection using the anti-ZYP1 antibody revealed clear signals on all chromosomes. The protocol can be adapted to other plant species with minor adjustments to the enzymatic treatment parameters. The high-quality well-spread pachytene chromosomes prepared by the developed protocol makes it easy the tracing of individual chromosomes from telomere to telomere, which facilitates the cytogenetic study of species with large genomes.

## Figures and Tables

**Table 1:** Mean SC and relative lengths of the ranked (1–8) bivalents of *Allium fistulosum*.

Chromosome	1	2	3	4	5	6	7	8	Total
Mean SC length ± SE * (μm)	121.93 ± 6.99	113.44 ± 6.31	108.22 ± 5.46	97.63 ± 4.94	86.72 ± 3.85	81.18 ± 3.94	77.64 ± 3.60	67.56 ± 3.08	754.32
Relative SC length	0.16	0.15	0.14	0.13	0.11	0.11	0.10	0.09	1.00

* — Standard error

## Data Availability

The original contributions presented in this study are included in the article/supplementary material. Further inquiries can be directed to the corresponding author.

## Supplementary Materials

The following supporting information can be downloaded at: https://www.mdpi.com/article/10.3390/mps8030054/s1.

## Abbreviations

The following abbreviations are used in this manuscript:

SC	Synaptonemal Complex
SE	Standard Error
SEM	Scanning Electron Microscopy
PMC	Pollen Mother Cell
