# Supplementary figures and images for: A Method of Well-Spread Pachytene Chromosome Preparations for Plant Species with Large Genomes Suitable for the Immunolocalization of Meiotic Proteins
Source: Methods Protoc. 2025 May 19;8(3):54. doi: 10.3390/mps8030054 (PMC12101289; doi:10.3390/mps8030054)

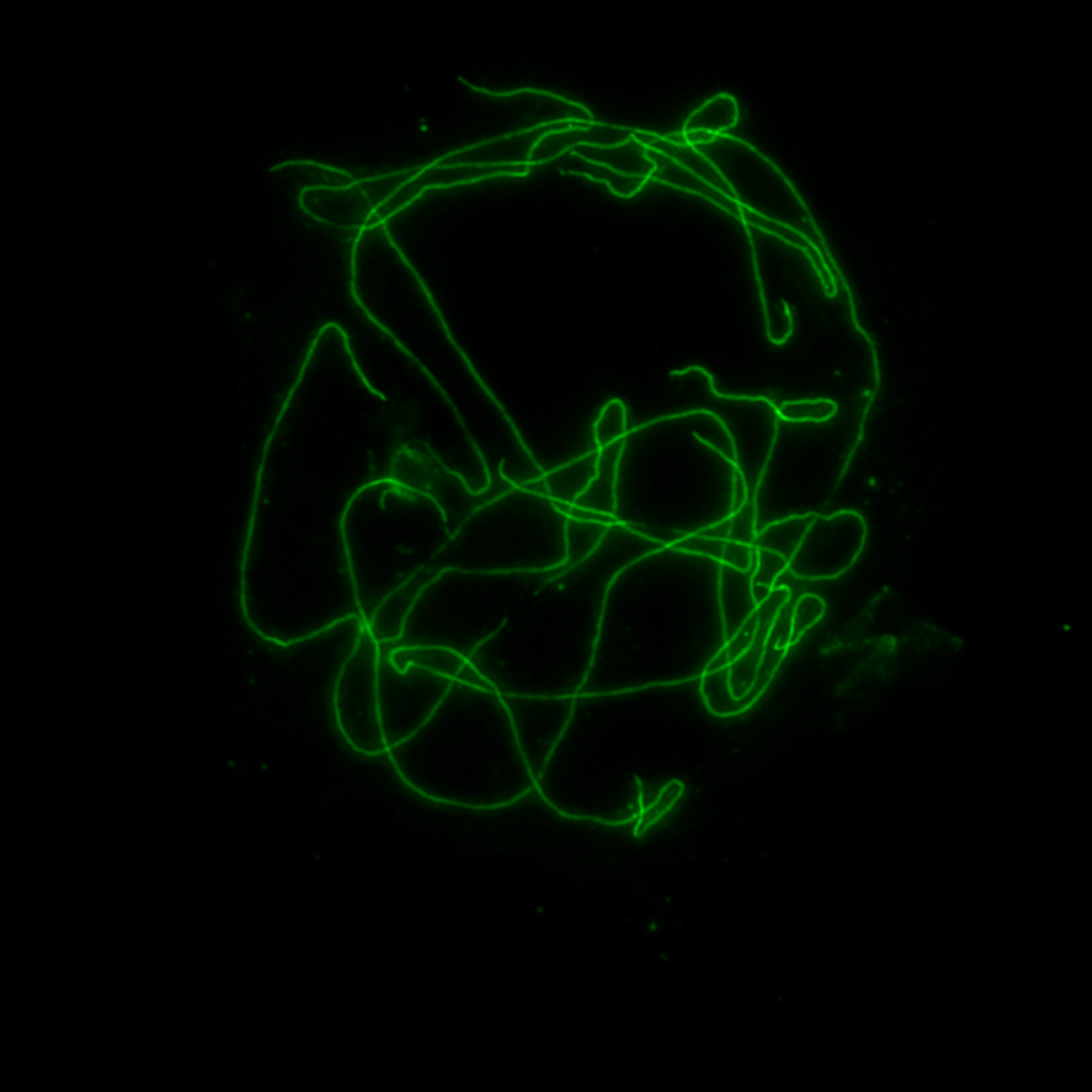

Supplement: Supplementary file 1 [file mps-08-00054-s001.zip › Fig2A.tif]

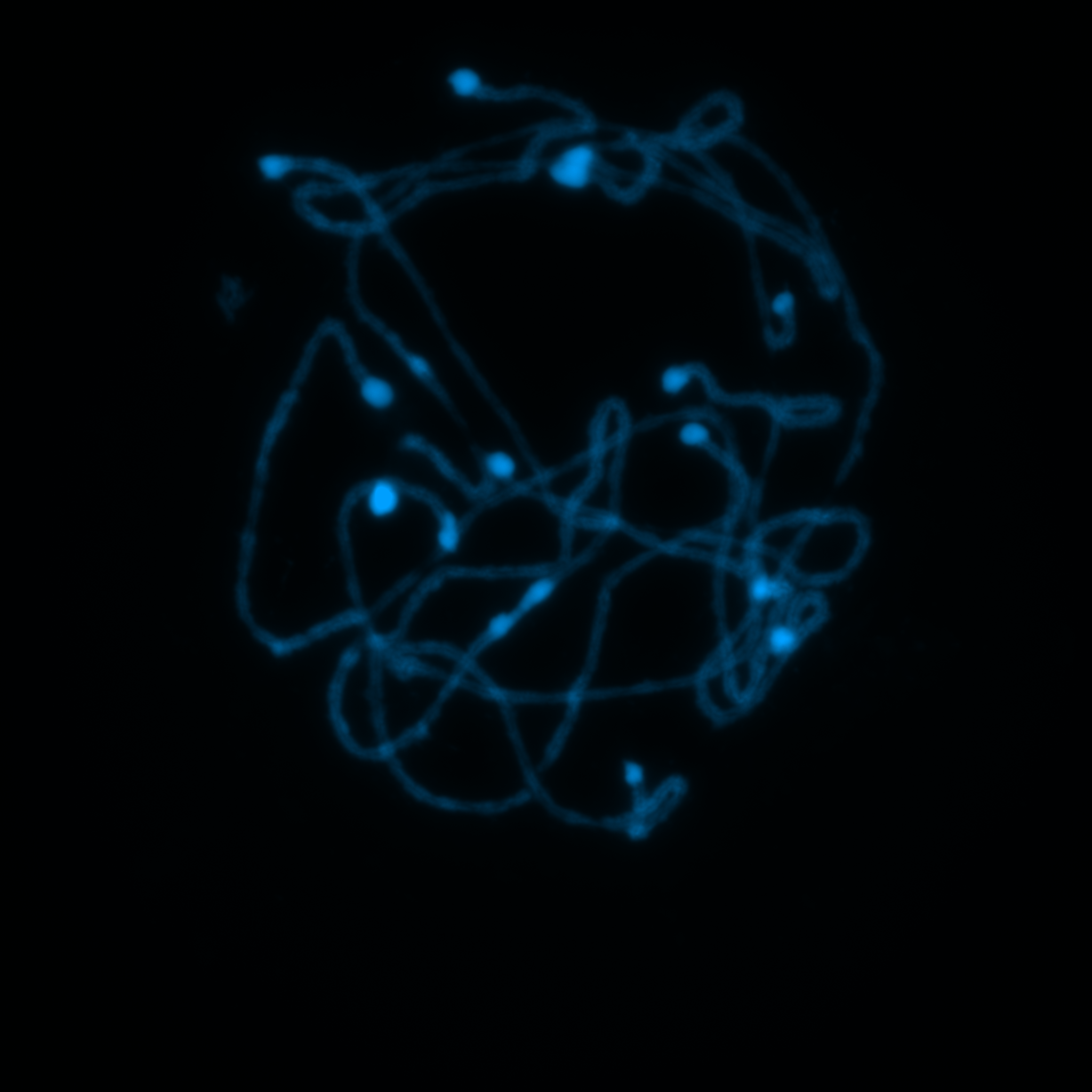

Supplement: Supplementary file 1 [file mps-08-00054-s001.zip › Fig2B.tif]

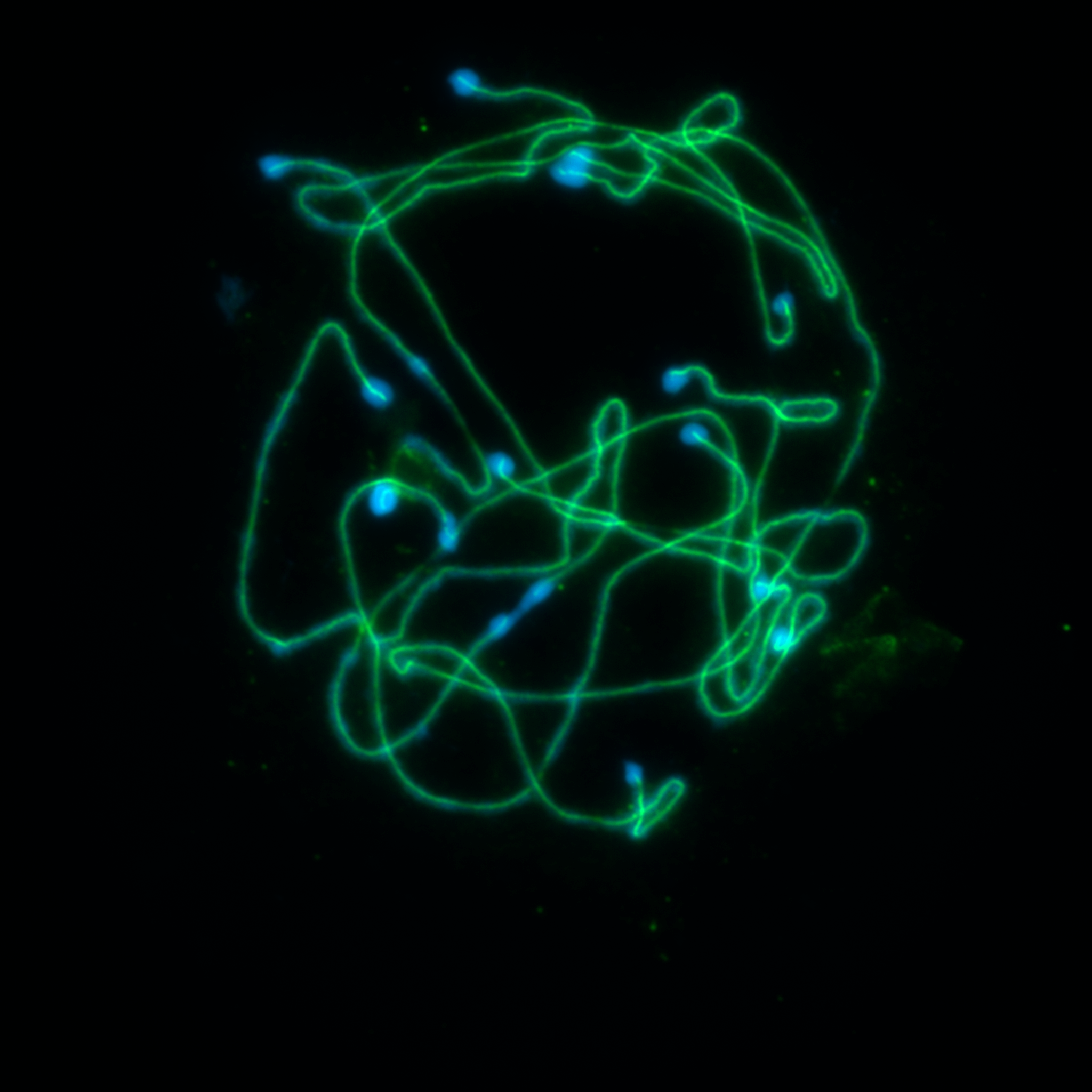

Supplement: Supplementary file 1 [file mps-08-00054-s001.zip › Fig2C.tif]

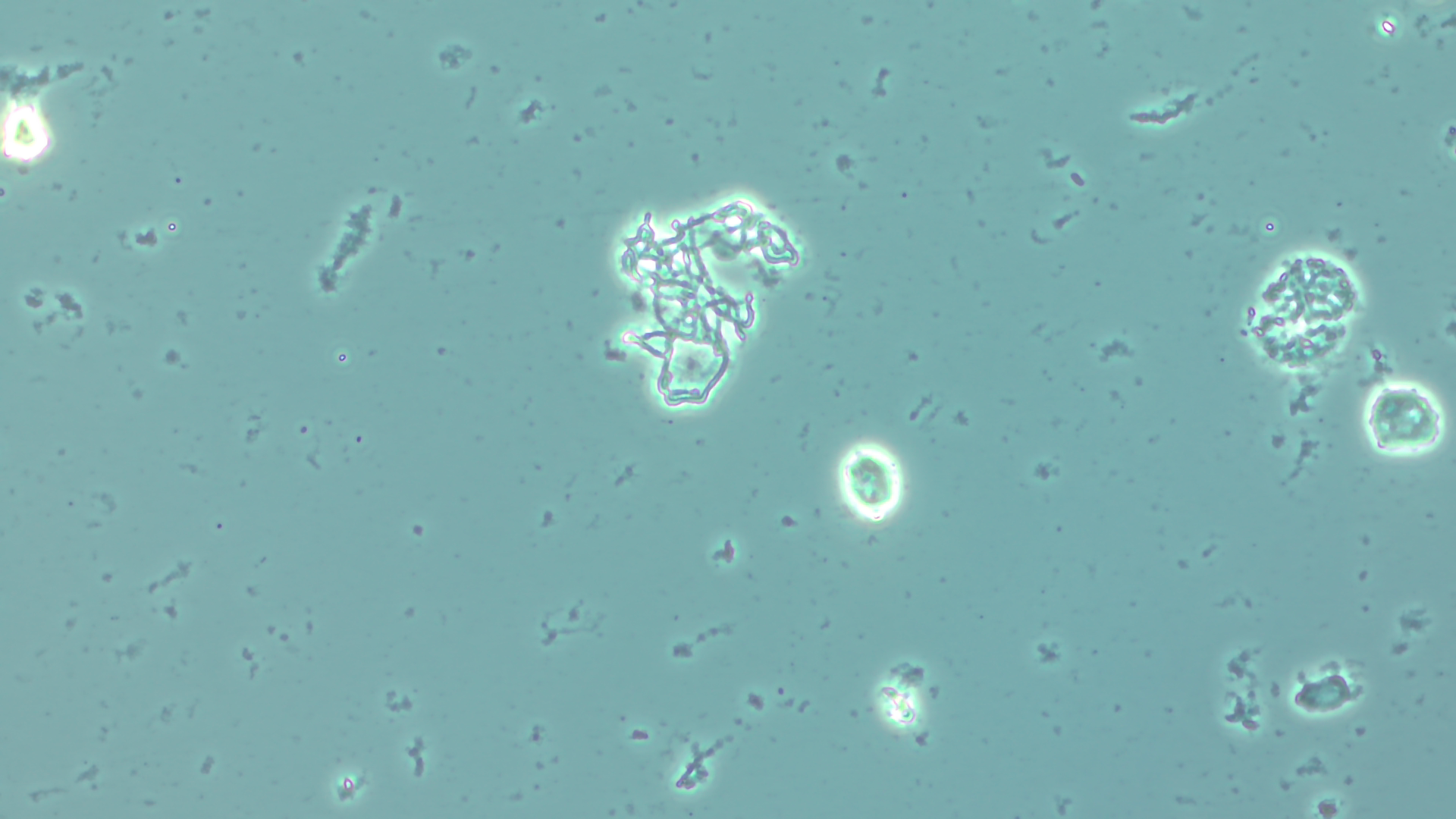

Supplement: Supplementary file 1 [file mps-08-00054-s001.zip › Fig3A.tif]

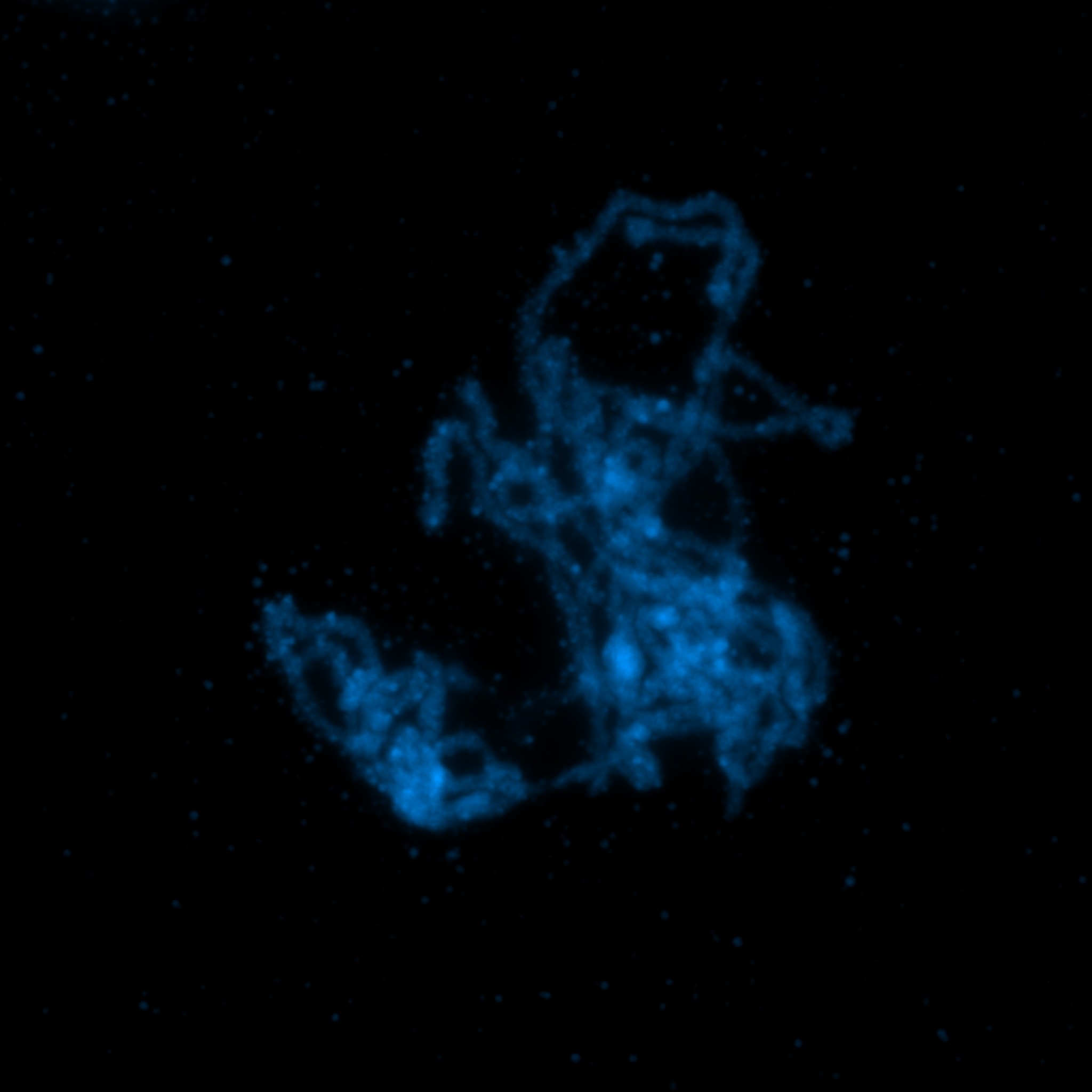

Supplement: Supplementary file 1 [file mps-08-00054-s001.zip › Fig3B.tif]

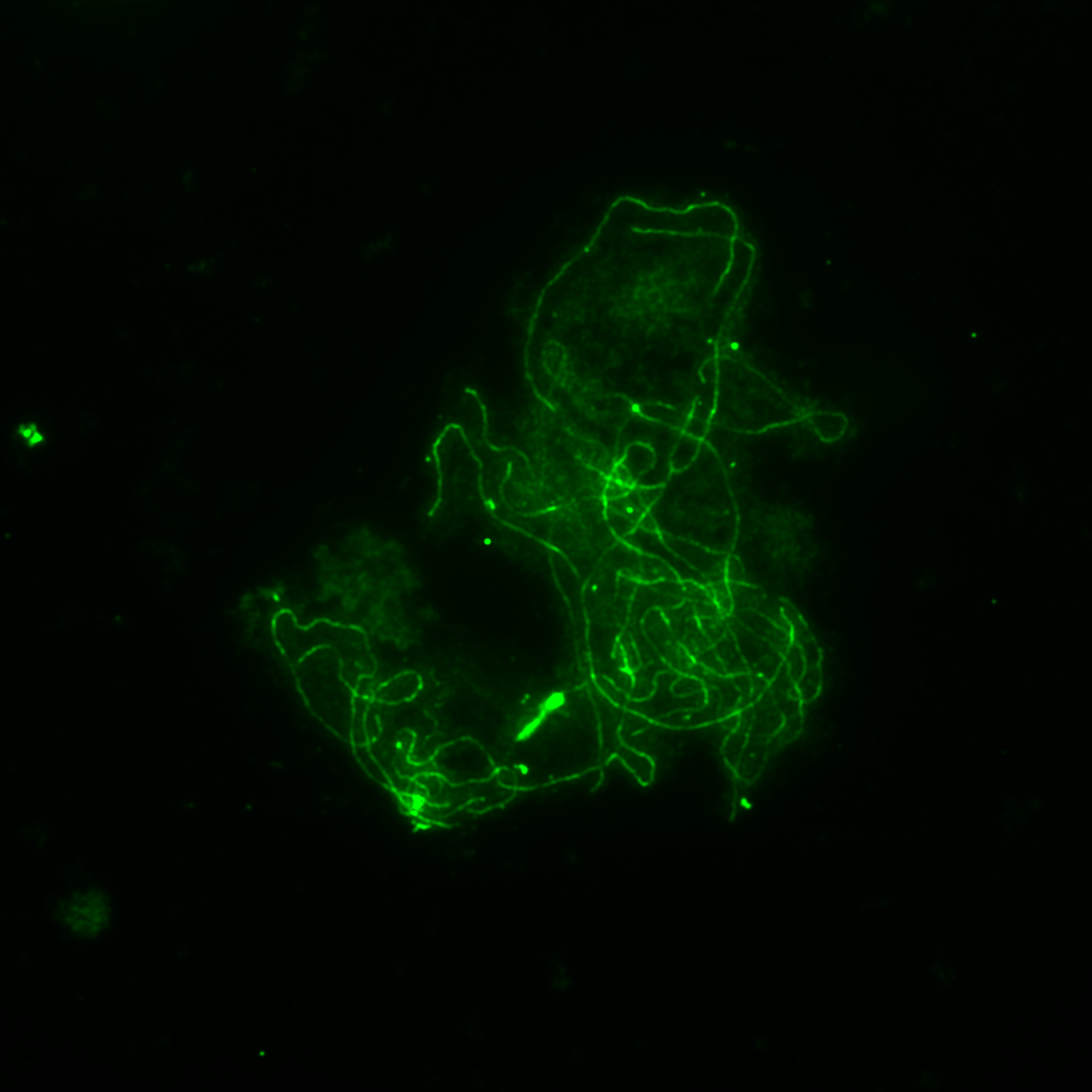

Supplement: Supplementary file 1 [file mps-08-00054-s001.zip › Fig3C.tif]

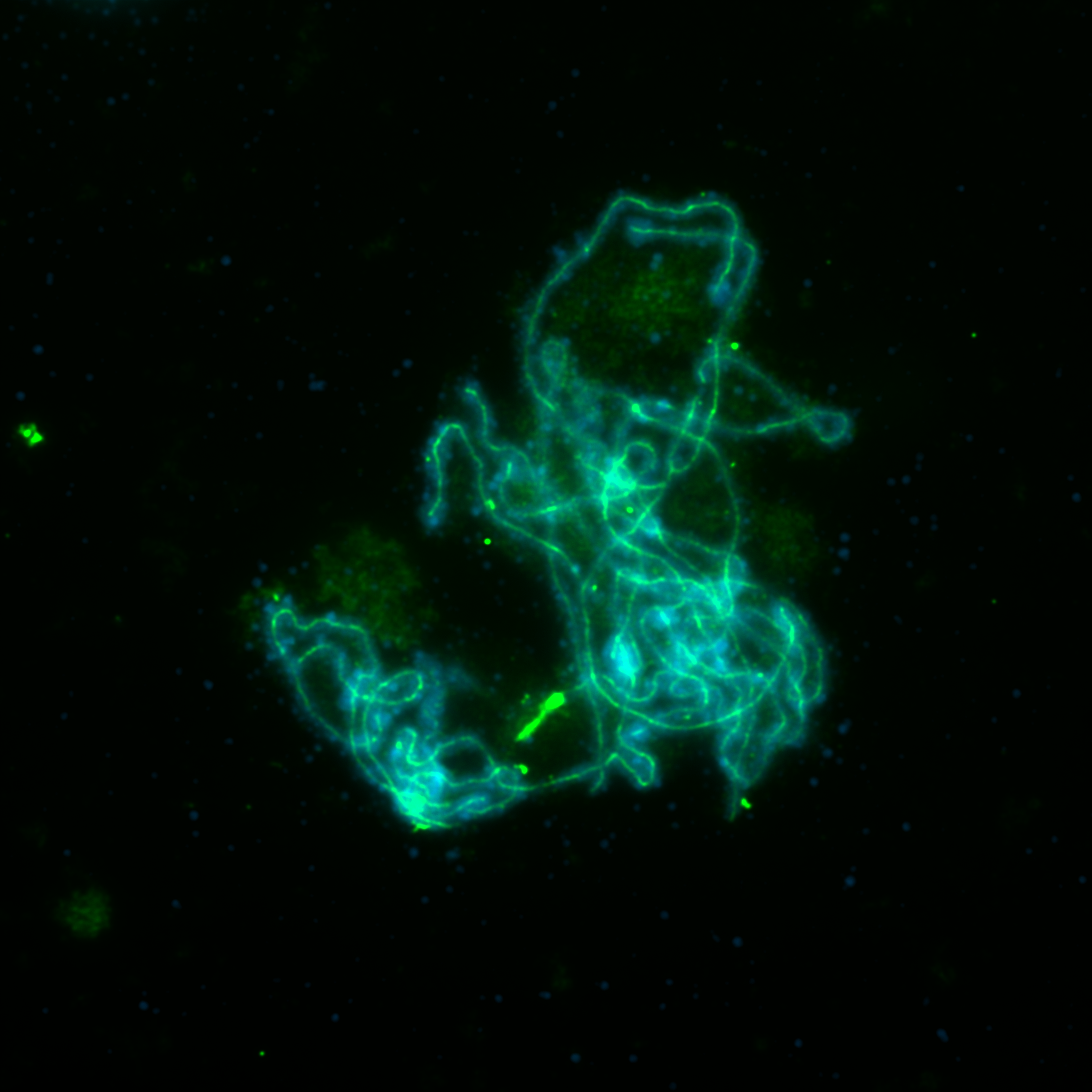

Supplement: Supplementary file 1 [file mps-08-00054-s001.zip › Fig3D.tif]

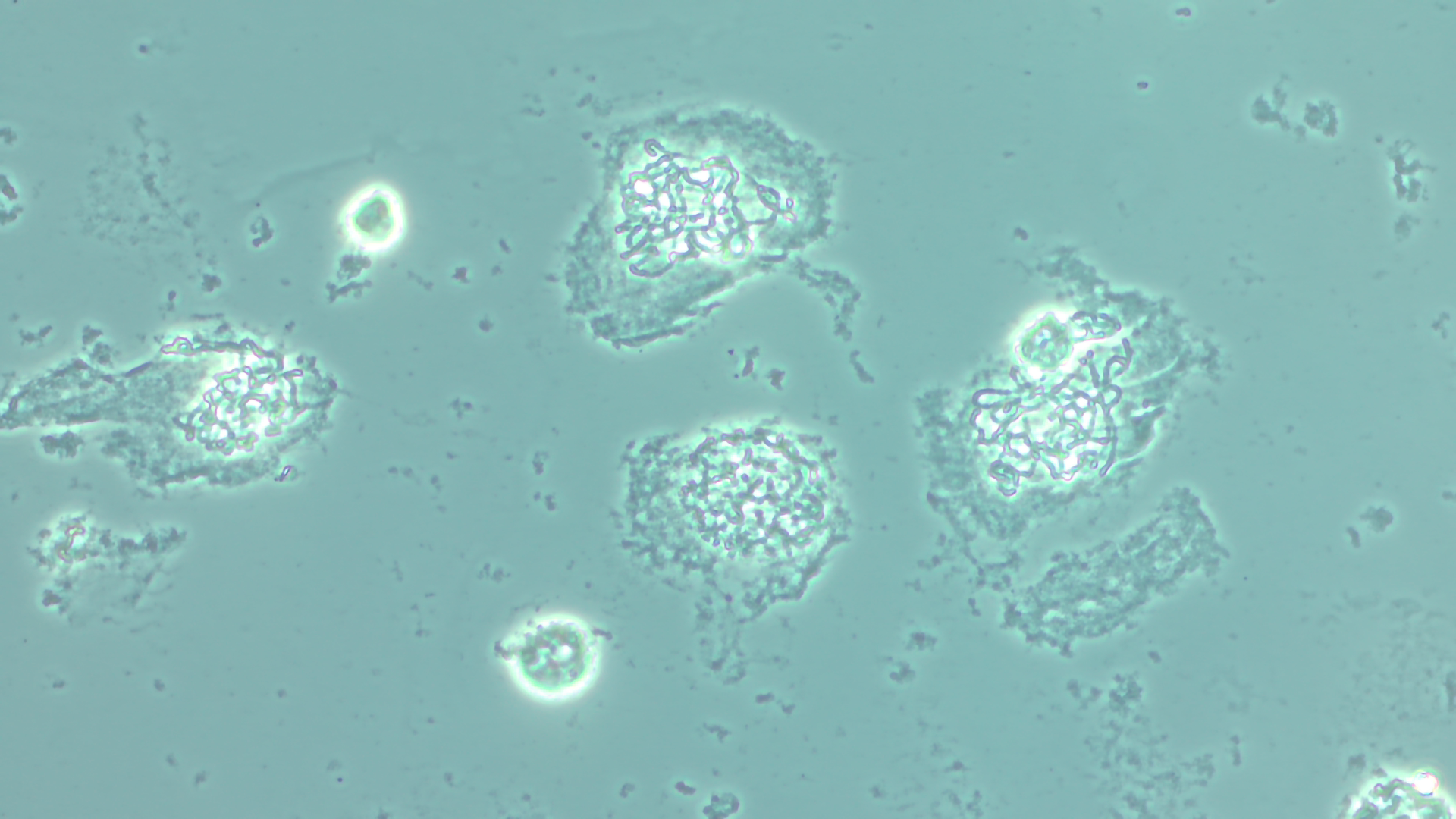

Supplement: Supplementary file 1 [file mps-08-00054-s001.zip › Fig3E.tif]

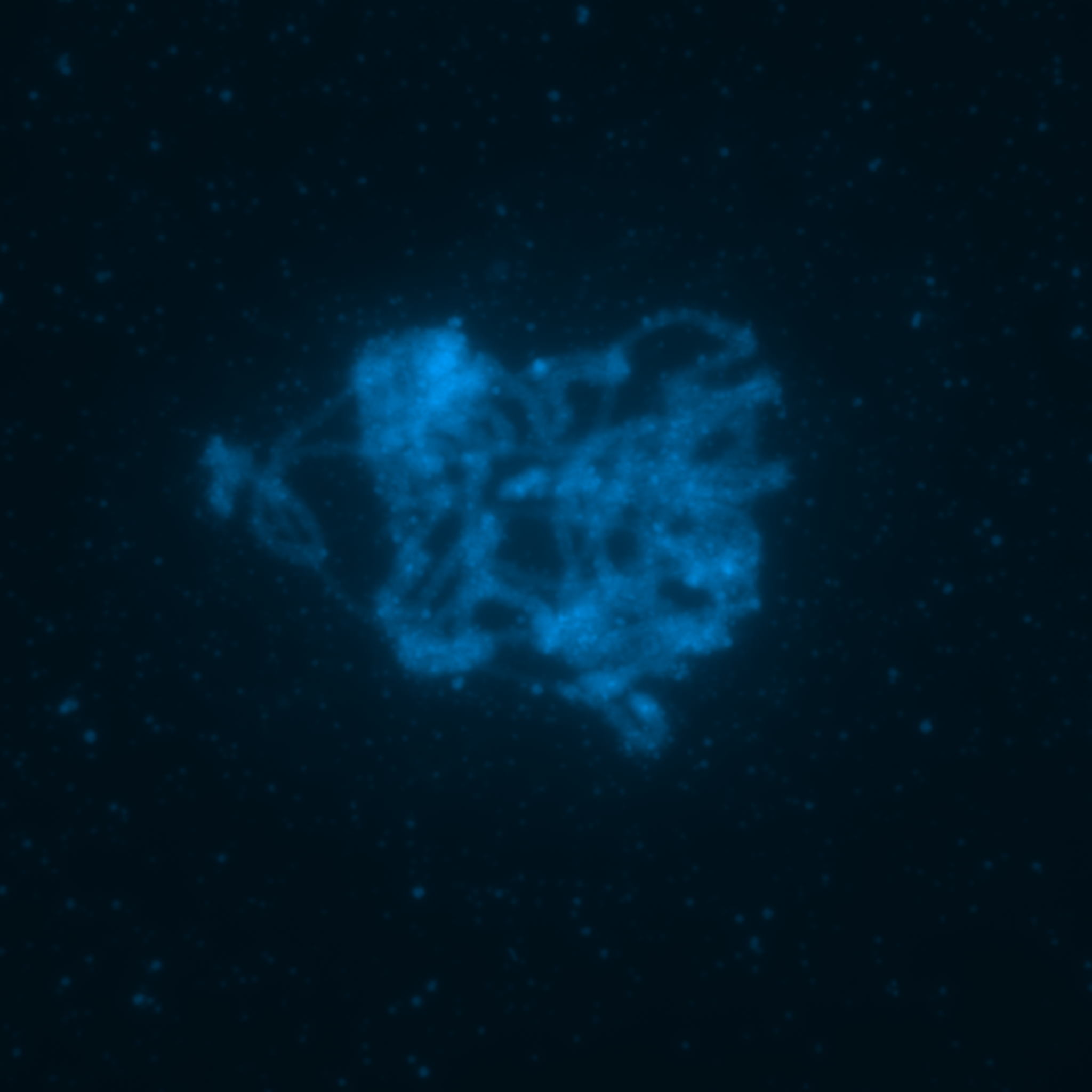

Supplement: Supplementary file 1 [file mps-08-00054-s001.zip › Fig3F.tif]

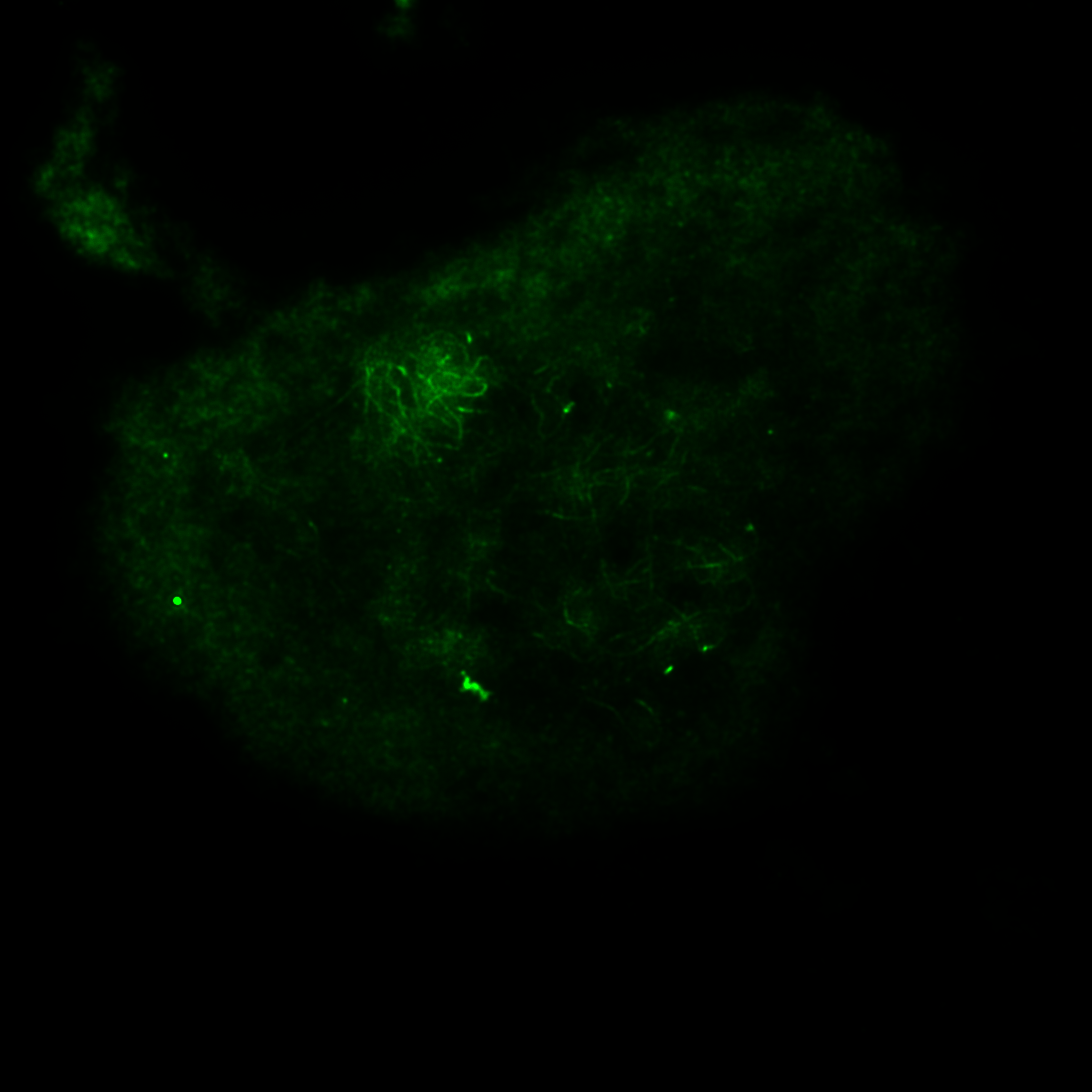

Supplement: Supplementary file 1 [file mps-08-00054-s001.zip › Fig3G.tif]

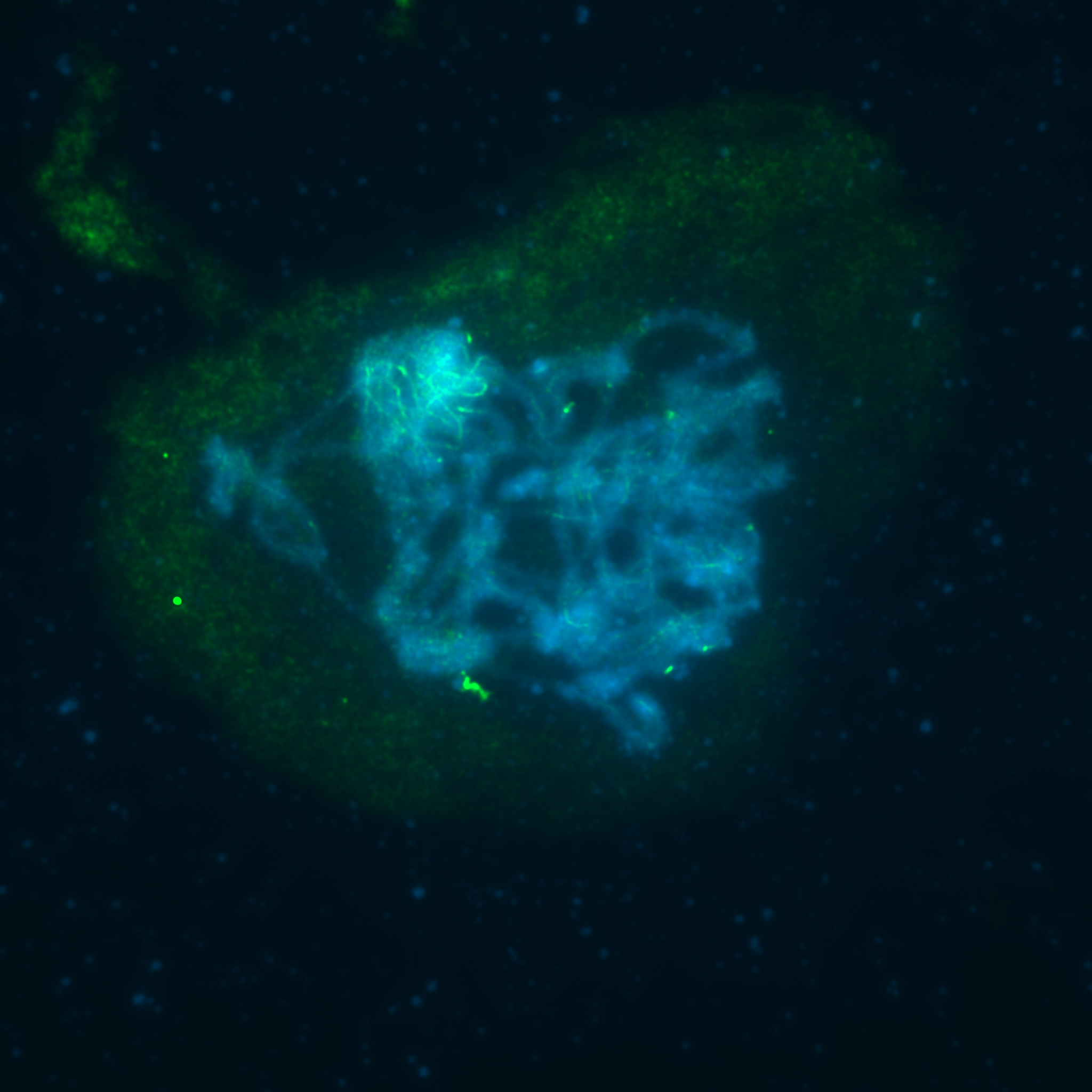

Supplement: Supplementary file 1 [file mps-08-00054-s001.zip › Fig3H.tif]

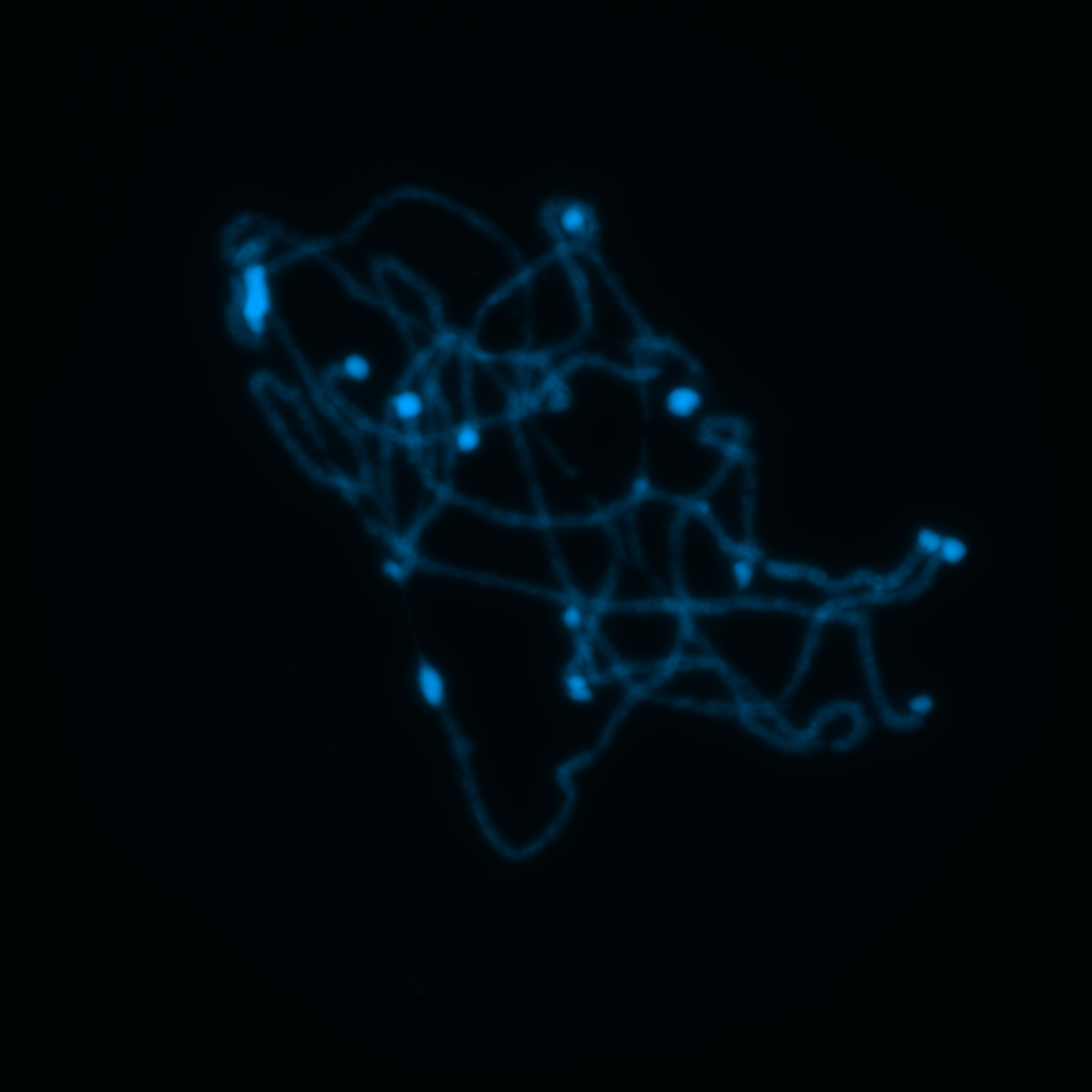

Supplement: Supplementary file 1 [file mps-08-00054-s001.zip › Fig4A.tif]

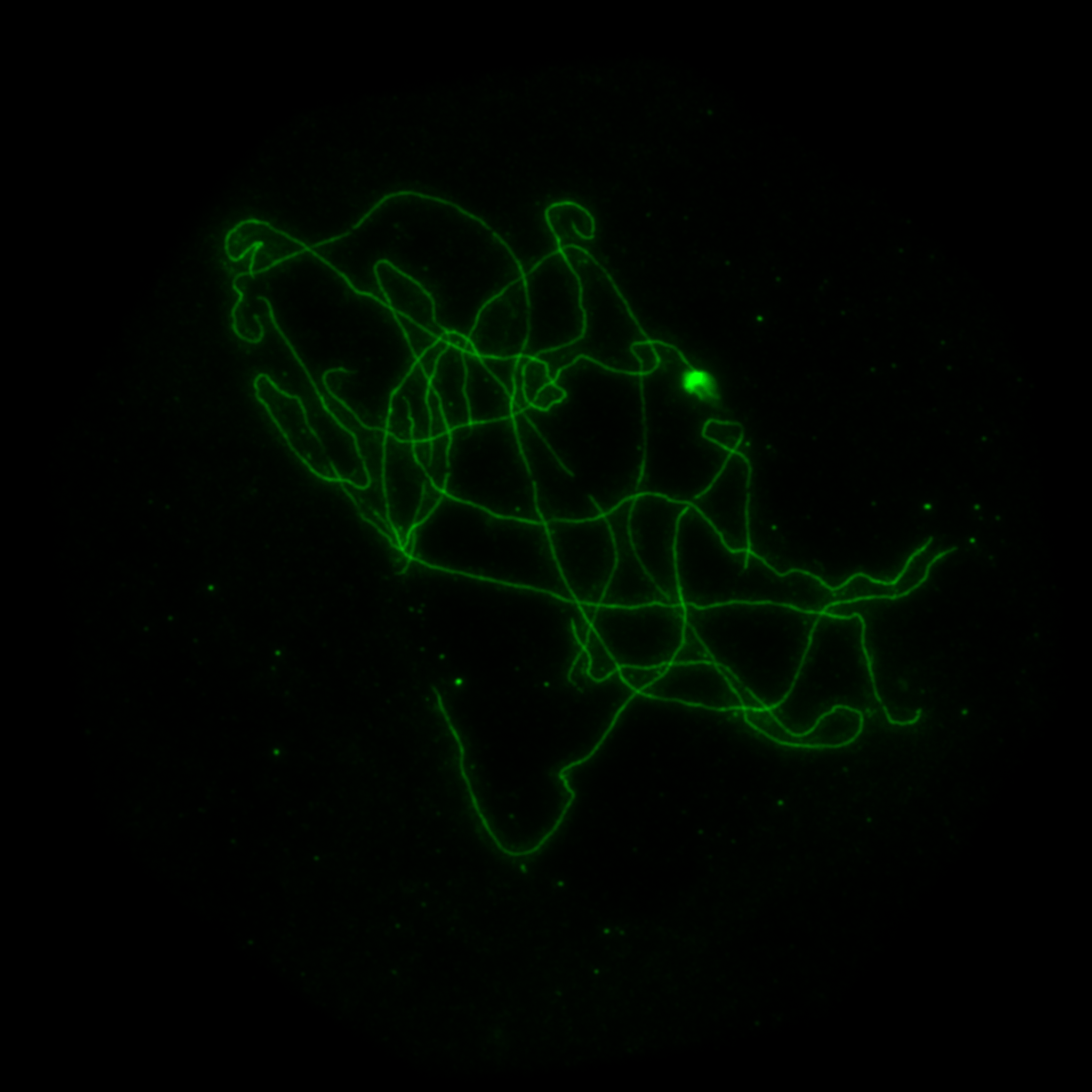

Supplement: Supplementary file 1 [file mps-08-00054-s001.zip › Fig4B.tif]

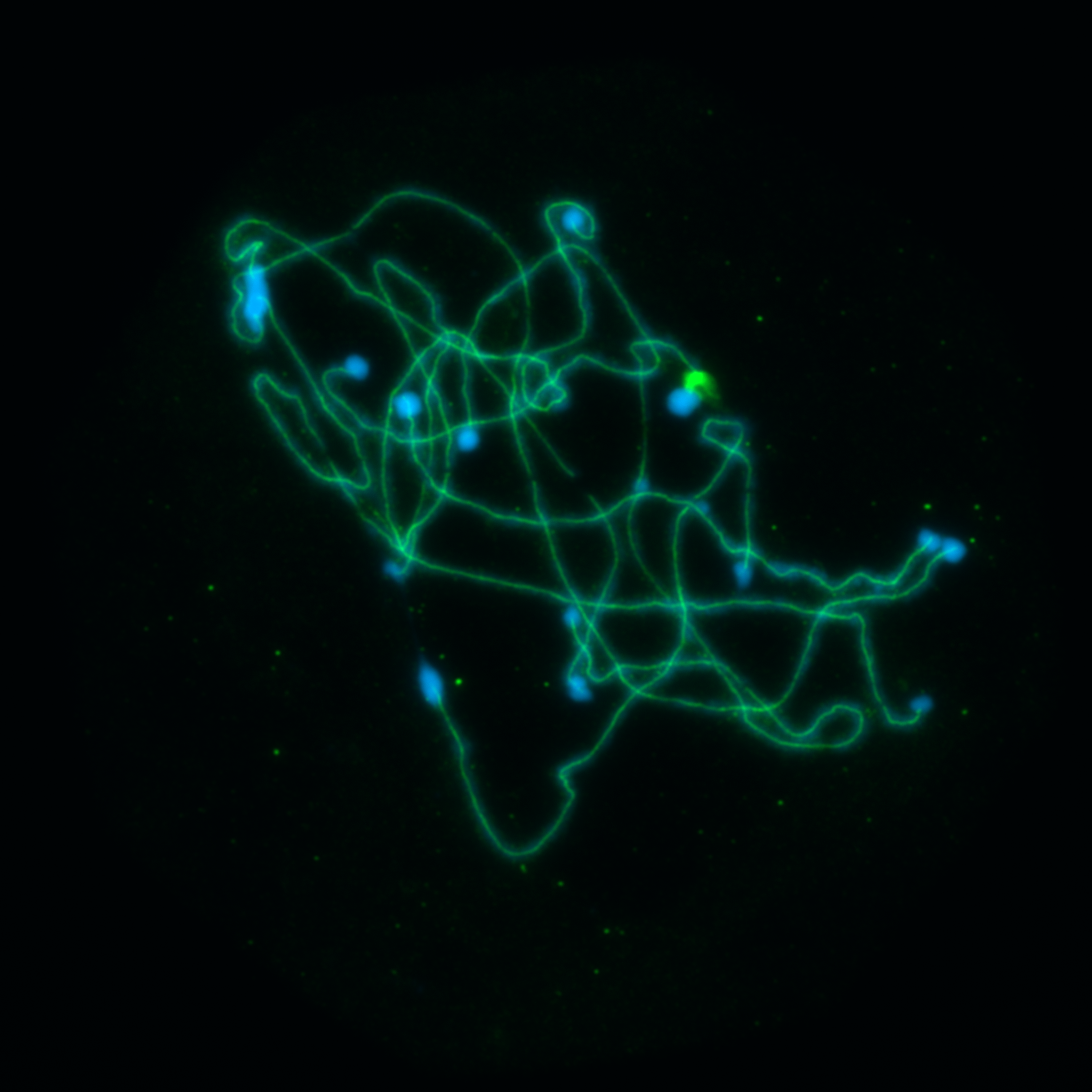

Supplement: Supplementary file 1 [file mps-08-00054-s001.zip › Fig4C.tif]

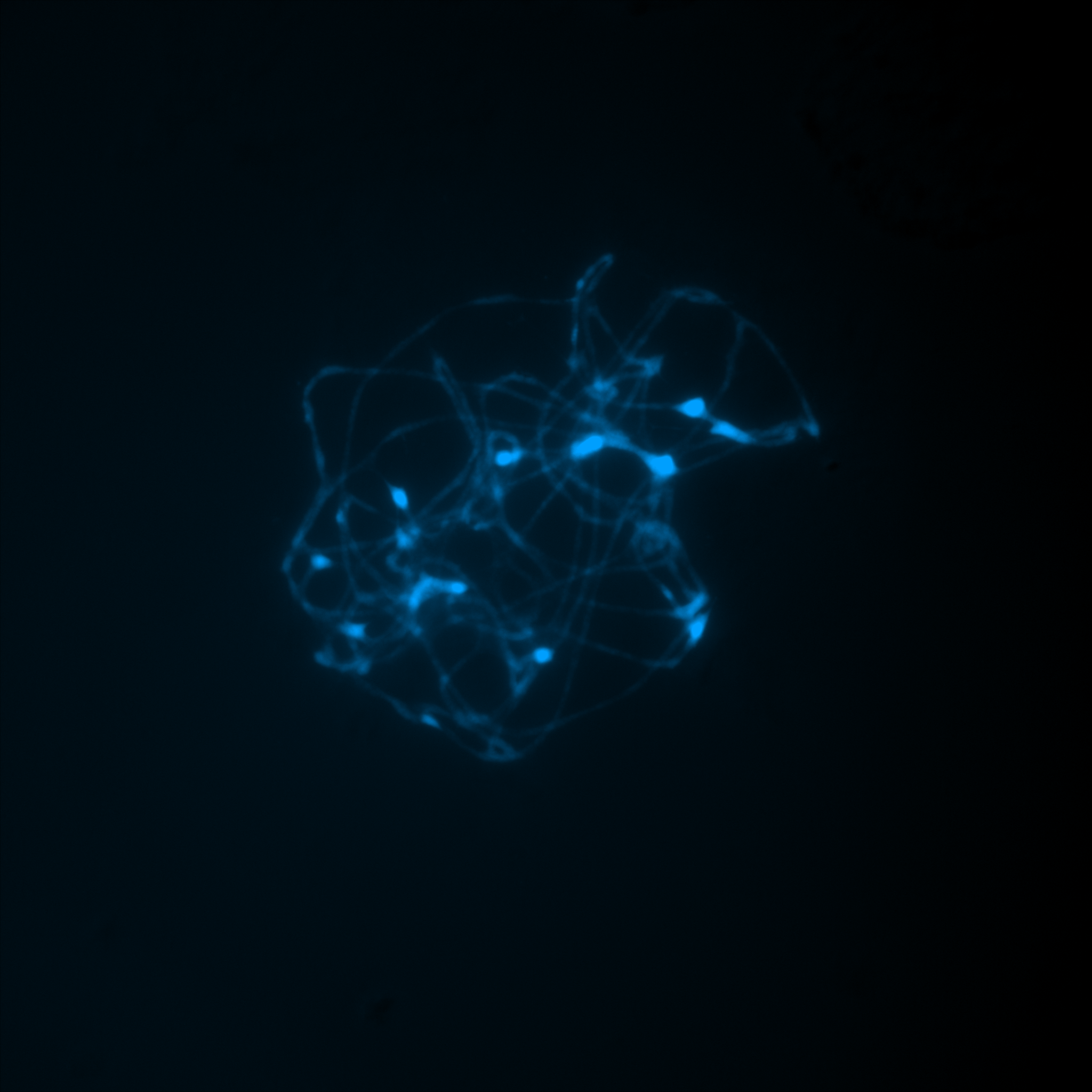

Supplement: Supplementary file 1 [file mps-08-00054-s001.zip › Fig4D.tif]

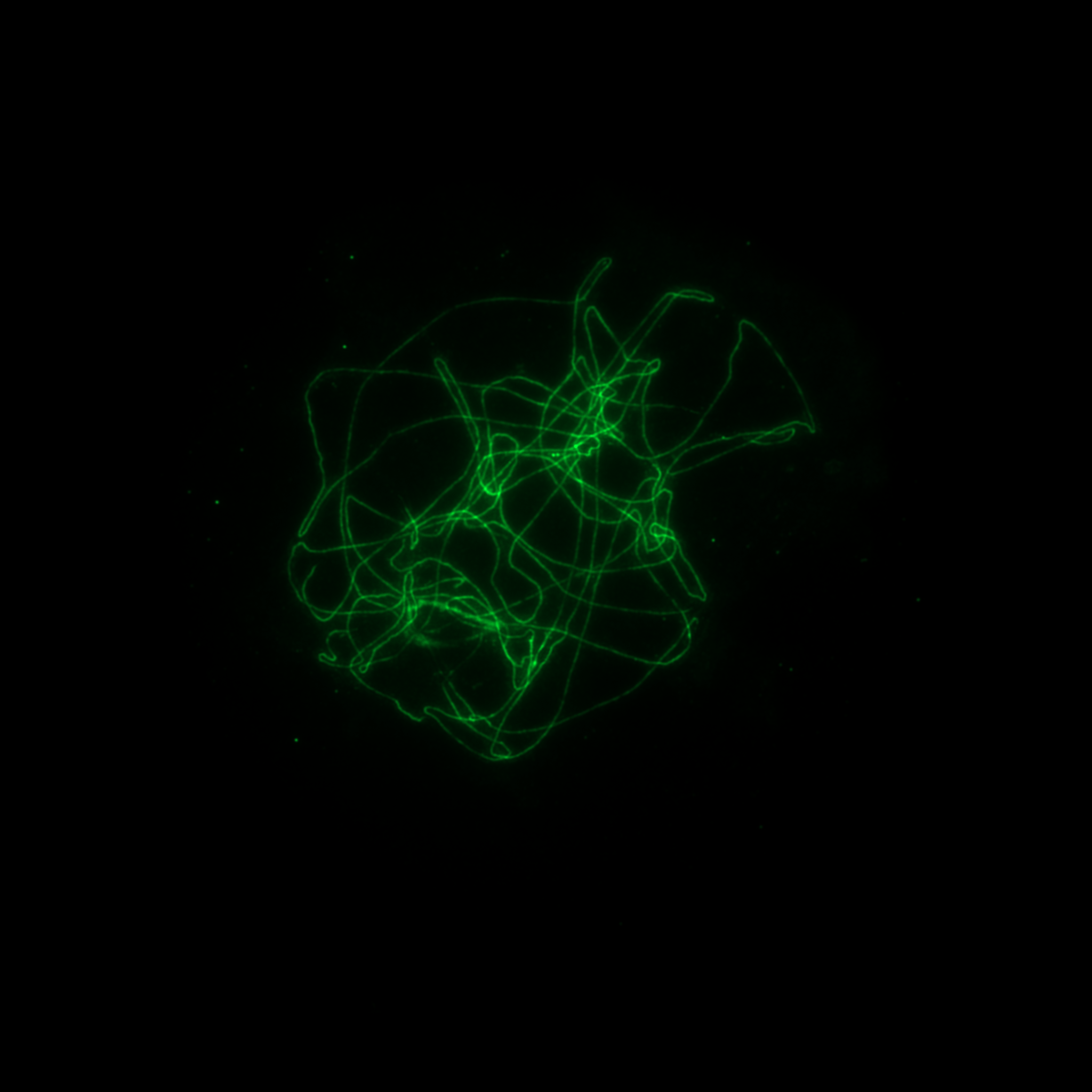

Supplement: Supplementary file 1 [file mps-08-00054-s001.zip › Fig4E.tif]

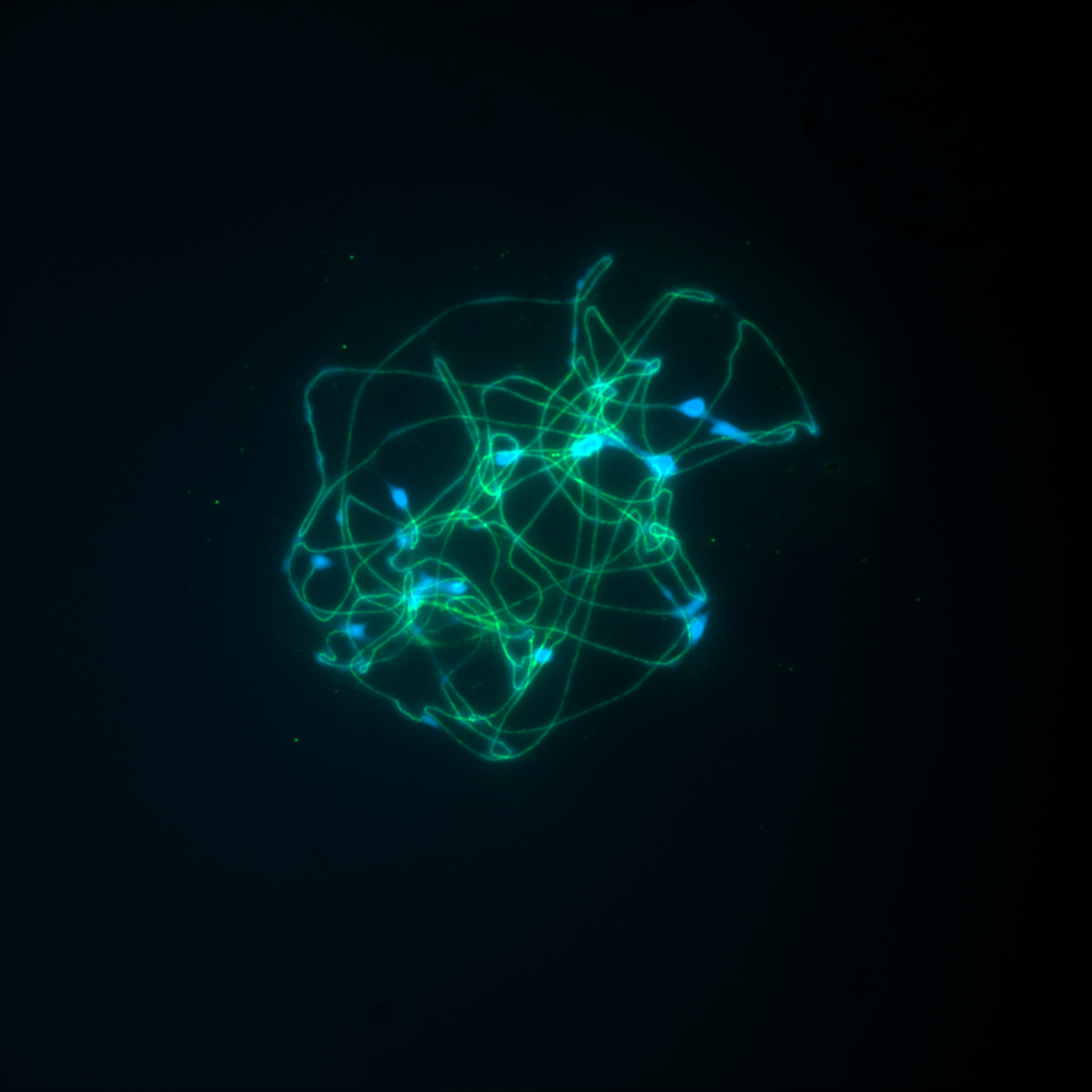

Supplement: Supplementary file 1 [file mps-08-00054-s001.zip › Fig4F.tif]

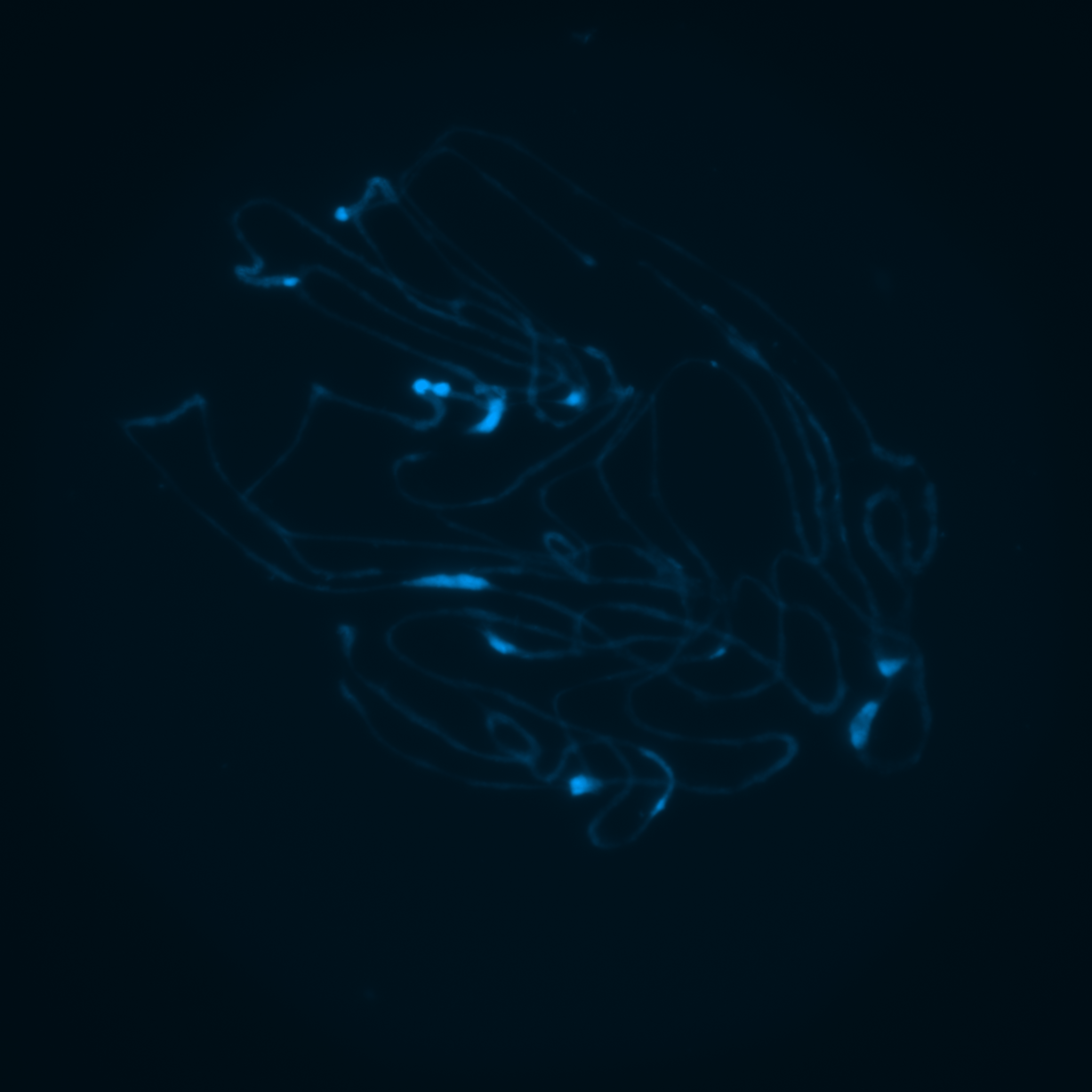

Supplement: Supplementary file 1 [file mps-08-00054-s001.zip › Fig4G.tif]

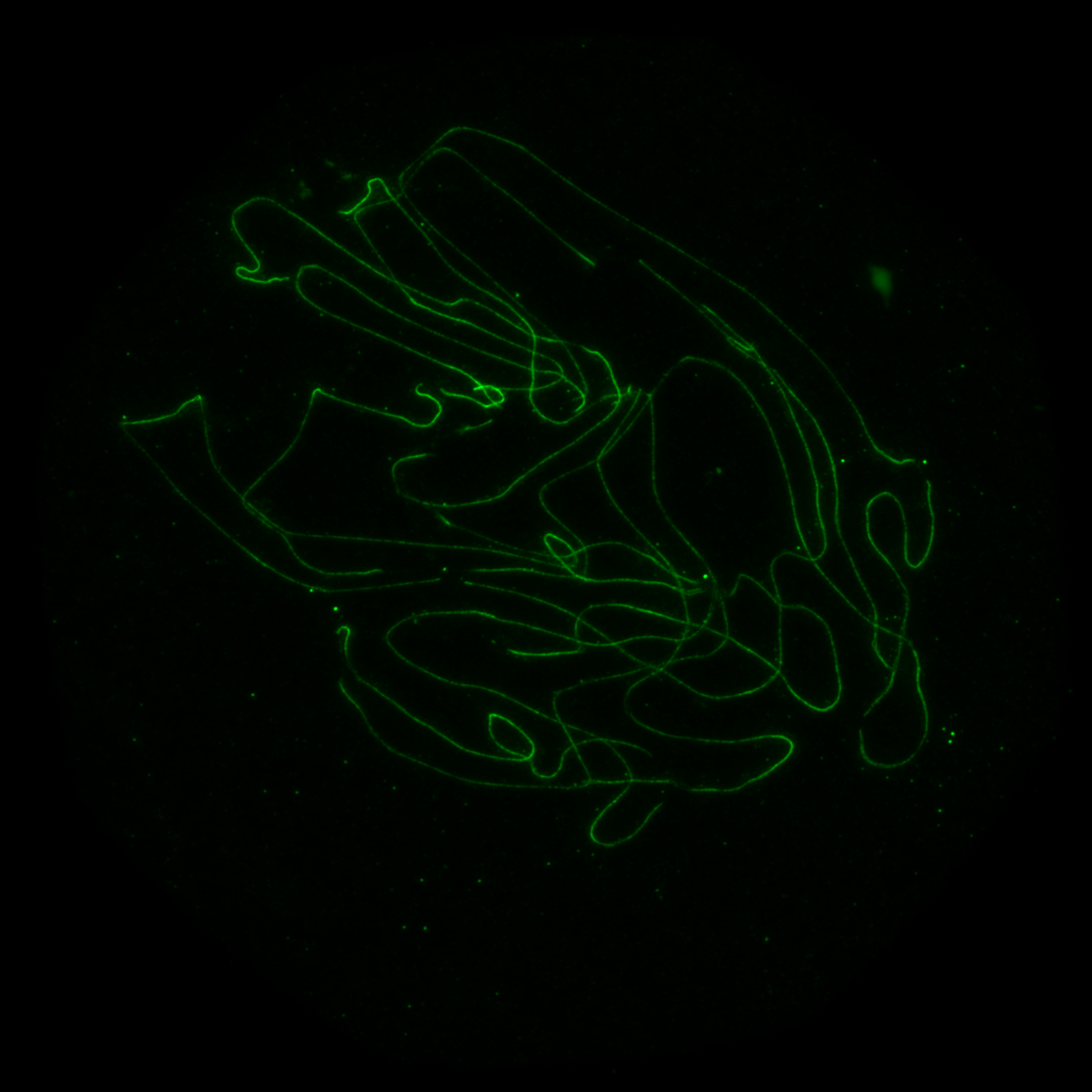

Supplement: Supplementary file 1 [file mps-08-00054-s001.zip › Fig4H.tif]

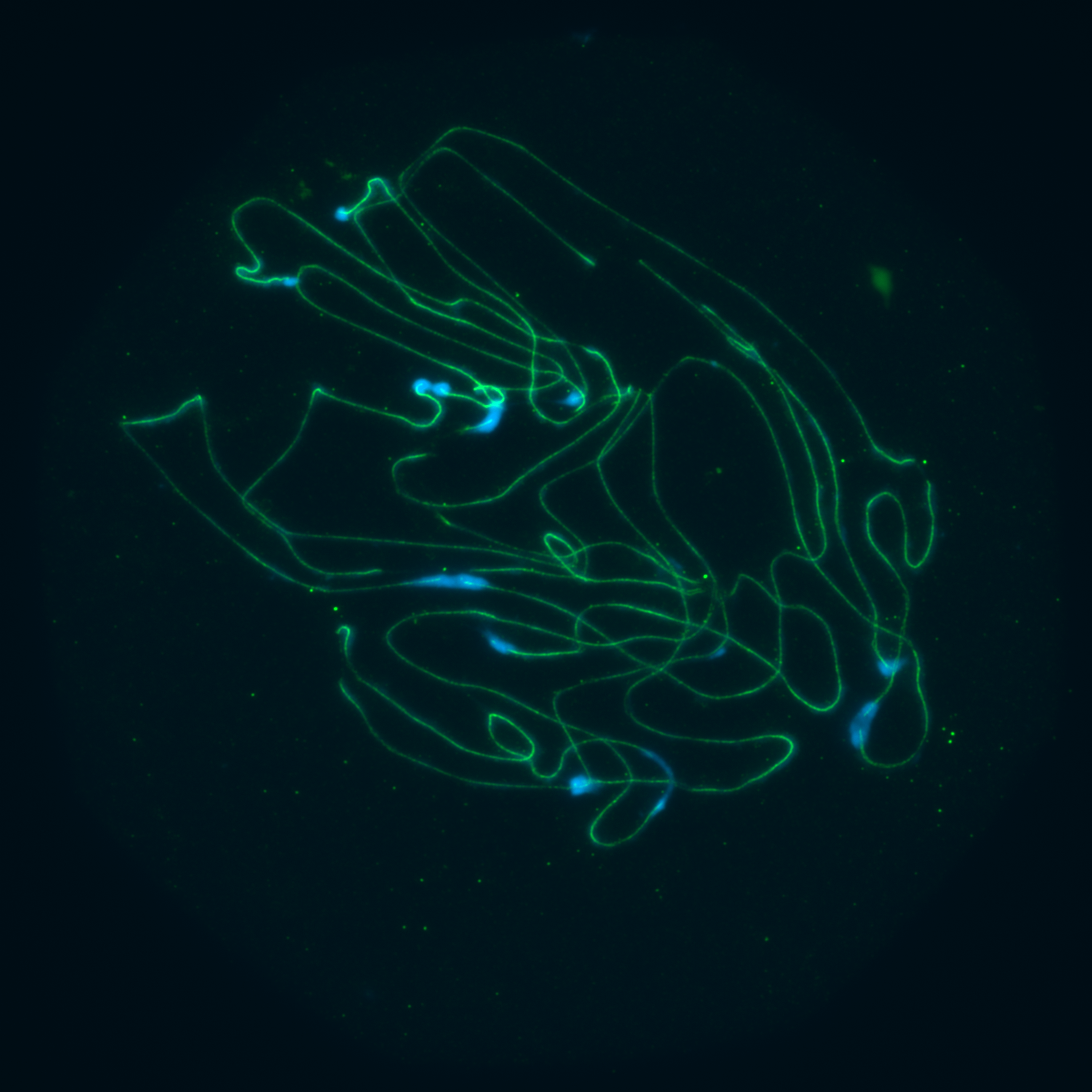

Supplement: Supplementary file 1 [file mps-08-00054-s001.zip › Fig4I.tif]
